# Dental Emergencies Management in COVID-19 Pandemic Peak: A Cohort Study

**DOI:** 10.1177/0022034521990314

**Published:** 2021-02-04

**Authors:** J. Beauquis, A.E. Petit, V. Michaux, V. Sagué, S. Henrard, J.G. Leprince

**Affiliations:** 1 Conservative Dentistry and Endodontics Department, Cliniques Universitaires St-Luc, UCLouvain, Brussels, Belgium; 2 DRIM Research Group, Advanced Drug Delivery and Biomaterials, Louvain Drug Research Institute (LDRI), UCLouvain, Brussels, Belgium; 3 Pharmacy Department, Cliniques Universitaires St-Luc, UCLouvain, Brussels, Belgium; 4 Clinical Pharmacy Research Group, Louvain Drug Research Institute (LDRI), UCLouvain, Brussels, Belgium; 5 Prosthetic Dentistry Department, Cliniques Universitaires St-Luc, UCLouvain, Brussels, Belgium; 6 Institute of Health and Society (IRSS), UCLouvain, Brussels, Belgium

**Keywords:** dental emergency treatment, dental care, pain relief, pain measurement, telemedicine, cross-contamination

## Abstract

Due to the global coronavirus disease 2019 pandemic, the high risk of cross-contamination and the overload of hospital facilities have resulted in a real urgency for restricting dental emergency patient flow. In this context, the objectives of the current work were to 1) measure the ability of a triage-based management strategy to limit patient admission and 2) evaluate the success rate of both on-site and remote emergency management regarding symptom relief and pain control over a 1-mo period. We included all patients contacting the dental medicine department for an emergency consultation during the lockdown, between April 1 and April 30, 2020 (*N* = 570). Following a telephone consultation and based on preestablished admission guidelines, a decision was made at baseline (T0) to either admit the patient for treatment or perform remote management by providing advice and/or drug prescription. Patients were then followed up systematically at 1 wk and 1 mo. Management failure was defined as the need for emergency admission for patients managed remotely since T0 and for new emergency admission for those admitted at T0. The global follow-up rate of patients with a complete data set was 91.4% (*N* = 521). Of included patients, 49.3% could be managed without admission for emergency reasons for 1 mo. The proportion of successful management was 71.8% and 90.2% at 1 mo for remote and on-site management, respectively. To conclude, the proposed triage-based emergency management strategy with systematic follow-up was a good compromise between limiting patient admission and ensuring effective symptom relief and pain control. The strategy can be useful in situations where regulation of the emergency patient flow is required.

## Introduction

In response to the initial outbreak of the coronavirus disease 2019 (COVID-19) pandemic, lockdowns and calls for community discipline were enforced worldwide to prevent the spread of severe acute respiratory syndrome coronavirus 2 (SARS-CoV-2), the virus responsible for the disease. All nonessential activities were suspended at the time, both in daily life and in the medical field. With the exception of COVID-19 treatments, medical activities were restricted to emergencies. The determination of an emergency situation was subject to interpretation and without formal guidelines or explicit criteria to support decision making. In this context, reconsideration of dental emergencies was required to ensure continuity of care while addressing the high risk of spreading COVID-19 (Bohmer et al. 2020; Cheng et al. 2020; Ganyani et al. 2020; Gudbjartsson et al. 2020).

It was essential to maintain a dental emergency activity, first to ensure appropriate treatment but also to inform and advise our patients. Dental infection is notably a common and potentially severe condition, arising mostly from dental caries (Robertson et al. 2015). The severe forms are therefore largely preventable if addressed appropriately and in a timely fashion. Moreover, orofacial pain is highly prevalent (Locker and Grushka 1987; Lipton et al. 1993; Macfarlane et al. 2002) and may reach high levels of intensity (Sharav et al. 1984; Seymour et al. 1985), similar to those observed in other painful diseases such as renal colic (>7/10 on a numerical rating scale) (Collaborative Group of the Spanish Society of Clinical Pharmacology 1991; Sasmaz and Kirpat 2019). This is further highlighted by the identification of dental pain as the major cause of acute medical admission due to unintentional paracetamol overdose (Siddique et al. 2015; O’Sullivan et al. 2018), which can lead to acute liver failure, a rare but potentially fatal adverse reaction. The high level and frequency of orofacial pain results in an elevated demand of emergency appointments in dental practice and in a certain proportion of general medical practices and emergency departments (Robertson et al. 2015). The high risk of cross-contamination and the overload of hospital facilities gave urgency in restricting dental emergency patient flow (Ather et al. 2020; Coulthard 2020; Meng, Hua, et al. 2020). Therefore, an efficient and rationalized dental emergency management strategy was required, including the implementation of teleconsultation and triaging. The strategy should identify the dental emergencies that cannot be effectively managed remotely (on-site management) and those emergencies considered eligible for remote management by means of remote advice and/or drug prescription. Effective symptom relief and pain control must, however, be verified in both situations.

Consequently, the objectives of the present work were to 1) measure the ability of a triage-based management strategy to limit patient admission over a 1-mo period and 2) evaluate the success rate of both on-site and remote emergency management over a 1-mo period.

## Methods

### Study Population, Design, and Triage Strategy

**Figure 1.**
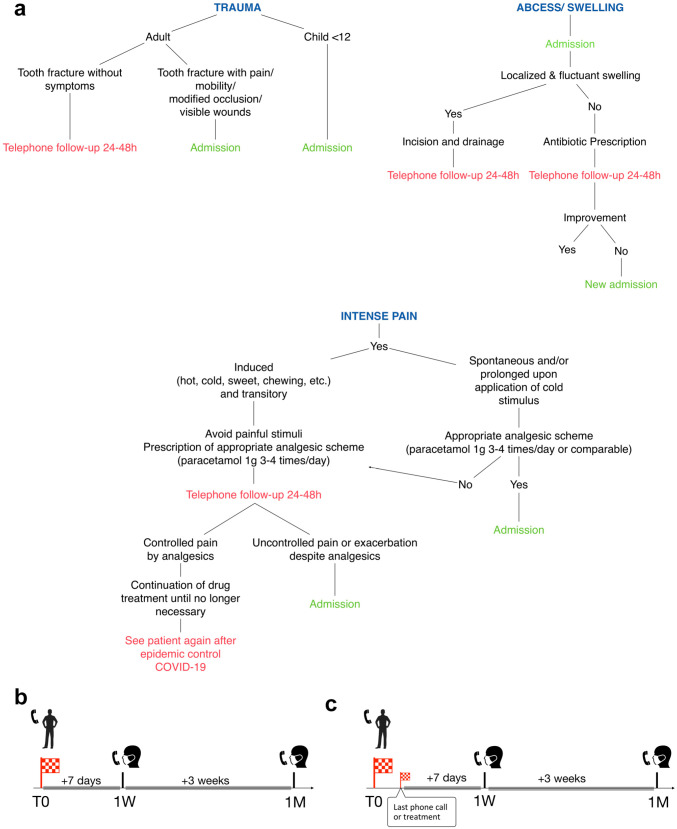
Guidelines established to support decision making (**a**) and patient recall pattern following the treatment decision at T0, (**b**) when no additional call or treatment was necessary at any time between the 3 timepoints, and (**c**) when additional advice or admission was needed. In that case, the 1W follow-up took place 7 d after the last contact, regardless of the number of calls.

This cohort study conformed to STROBE guidelines and included all patients contacting the dental medicine department of the Cliniques universitaires St Luc (Brussels, Belgium) for an emergency consultation between April 1 and April 30, 2020 (inclusive). The COVID-19 data related to this period and location are presented in the Appendix. For all patients, a triage strategy was applied via telephone by the practitioners to determine if the patient required admission based on the admission guidelines (Fig. 1a). A decision was made at T0 to either manage the patient remotely (remote management group) or to admit the patient for on-site treatment (on-site management group). If the patient directly attended the hospital medical emergency department, similar teleconsultation was performed through an internal line within the central hall of the hospital. Practitioners (*n* = 44) belonging to pediatric, prosthetic, and conservative dentistry and endodontics were involved in emergency management over the lockdown period. There was a daily assignment of 7 practitioners, all sharing treatment, telephone triage duties (random assignment, including for pediatric cases), and follow-up calls 7 d a week from 09:00 to 20:00.

The admission guidelines were established during the first 2 wk of the Belgian lockdown (from March 18 to March 31, 2020; inclusive), based on best-practice recommendations (Dorn and Cheung 2016) and validated by a consensus group involving all available practitioners. Briefly, admission at the time of the first telephone contact (= T0) was reserved for emergencies that could not be effectively managed remotely (i.e., dental trauma, intense pain, and abscess) since these were considered as severely affecting patient quality of life and/or presenting a high risk of evolution to severe forms of disease. By exclusion, all other indications were categorized as remote management at T0. The decision to admit all children <12 y in case of trauma was based upon the difficulty to evaluate the severity of their condition by teleconsultation. Occasionally, the teleconsultation was optimized by suggesting that patients send pictures electronically; however, this was not systematically measured.

Our admission guidelines were not binding but indicative, leaving a certain freedom of interpretation to practitioners. The guideline document (Fig. 1a) was available to each practitioner and posted near each telephone set.

### Patient Recall

Following the treatment decision at T0, a follow-up telephone call was conducted systematically at 1 wk (1W) and 1 mo (1M) (Fig. 1b). The patients were contacted by 1 dentist on the treatment team on duty who typically was not the practitioner who originally spoke with the patient. For some patients following the baseline decision making, the short-term follow-up (<1W) necessitated additional advice or admission. The 1-wk follow-up period was then considered to start after the last phone call or treatment (Fig. 1c). The 1-mo follow-up was performed precisely 3 wk after the 1W follow-up.

Of the 570 patients included at T0, we excluded those showing inappropriate behavior at T0 (e.g., aggressiveness upon nonadmission decision), those who did not attend despite an admission decision at T0, and those with lack of follow-up at 1W or 1M.

### Data Collection

Systematic forms were used to collect the data during each phone call.

Patient administrative data, symptoms, pain intensity on a 0 to 10 numerical rating scale, medication use, telediagnosis, nonpharmacologic and pharmacologic advice if provided, and the seniority of the dentist in charge of teleconsultation were collected at T0. For admitted patients, the clinical diagnosis and type of treatment provided were also recorded. Pain scale assessment was not collected for children aged <12 y.

At 1W, the follow-up teleconsultation aimed to evaluate the efficacy of the treatment prescribed at baseline (T0) (advice and/or drug prescription and/or local treatment). Data collection included the evolution of symptoms compared with the baseline, pain intensity, nonpharmacologic and pharmacologic advice (if provided), or whether the patient had contacted or visited another dentist.

At 1M, the follow-up teleconsultation aimed to evaluate the stability of the results achieved at 1W with the addition of COVID-19 status. The latter consisted of 3 categories: no suspicion or diagnosis, suspicion (with or without medical advice, test not carried out or negative), or confirmed (by reverse transcription polymerase chain reaction [RT-PCR], serology, or chest computed tomography [CT] scan). If an additional advice or treatment was provided at, or between, 1W and 1M, the reasons were also recorded, that is, whether it was due to the resumption of activity of the general dental practitioner (May 4, 2020, in Belgium) or an emergency visit due to escalating symptoms.

The various types of emergency diagnoses were grouped by category for descriptive statistics and statistical analysis (i.e., pulp pathologies, periapical pathologies, traumas, other types of inflammation and pain, and other emergencies) (Appendix).

The medications were grouped by 2 categories: 1) antibiotics (Anatomical Therapeutic Chemical [ATC] codes J01) and 2) analgesics (ATC codes N02A and N02B) and nonsteroidal anti-inflammatory drugs (NSAIDs) (ATC codes M01A) (separate and combined use).

Three categories of dentist seniority were chosen according to the number of years postgraduation (1 y, 2 to 3 y and >3 y).

### Outcome Measurement

Within the remote management group (patients not admitted at T0), management success was defined as nonemergency admission at any time (≤1M). Within the on-site management group (patients admitted at T0), management success was defined as no further emergency admission (≤1M). An admission to a different treatment facility for emergency reasons was considered in the same way as one in our institution (i.e., as management failure).

The first main outcome was the ability to limit patient admission, quantified as the proportion of the included patients who could be successfully managed remotely without the need for emergency admission (equivalent to the ratio of the number of patients successfully managed remotely and the total number of included patients) over the follow-up period of 1 mo.

The second main outcome was the success rate within each group (on-site and remote) over the follow-up period of 1 mo.

Secondary outcomes were also considered. First, the evolution of symptomatology and pain intensity (from T0 to 1W and from 1W to 1M) was evaluated in both groups for successful cases that did not require any additional treatment or advice at any time. Second, the risk of COVID-19 infection within the month following emergency dental treatment was evaluated for patients and practitioners involved in the study.

### Statistical Analysis

Continuous variables were compared between groups using a Kruskal-Wallis test and categorical variables using Pearson χ² or Fisher-Freeman-Halton test. The Wilcoxon signed-rank test was used to compare pain score evolution. Factors associated with admission at T0 and management failure were assessed using a binary logistic regression. A *P* value <0.05 was considered statistically significant. A more detailed description of statistical analysis can be found in the Appendix.

## Results

### Limitation of Patient Admission

**Figure 2.**
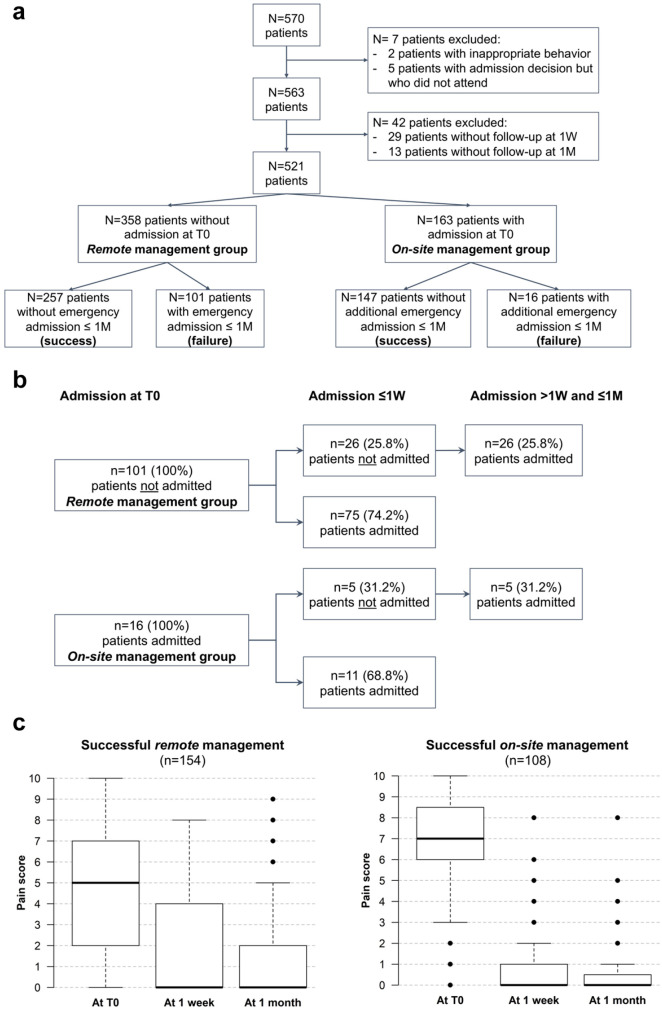
Global patient flowchart (**a**), flowchart of patient chronology of admission for cases classified as management failures in both groups (**b**), and boxplots of the evolution of pain score at 1 wk (1W) and 1 mo (1M) of successful management cases that did not require any additional treatment or advice (**c**). The reduction of pain scores between timepoints was significant in all cases (*P* < 0.001) except between 1W and 1M for on-site management (*P* = 0.053).

Remote management was decided for approximately two-thirds of the included patients, and the remaining one-third was admitted at T0 (Fig. 2a). Approximately half of the included patients (49.3%) were managed without admission for emergency reasons for 1 mo. The investigated variables and their distribution in the whole sample and according to the management outcome are listed in Table 1. Due to the limited number of management failures in the on-site group (*n* = 16), the management failures of both groups were grouped together for this analysis.

**Table 1.** Patients’ Characteristics at Baseline (T0) According to the Patient’s Management Outcome and COVID-19 Diagnosis at 1-mo Follow-up (*N* = 521).

Variables	Total (*N* = 521) Median (P_25_; P_75_) or *n* (%)	ManagementSuccess within Remote Management Group (*n* = 257), Median (P_25_; P_75_) or *n* (%)	ManagementSuccess within On-SiteManagement Group (*n* = 147),Median (P_25_; P_75_)or *n* (%)	Management Failure in Both Remote and On-Site Management Groups (*n* = 117), Median (P_25_; P_75_) or n (%)	*P* Value
Age, y	36.6 (25.5; 51.7)	36.7 (24.1; 57.3)	33.5 (21.6; 45.0)	40.9 (32.3; 50.0)	0.002
Age, y					<0.001
<12 y	64 (12.3)	41 (16)	20 (13.6)	3 (2.6)	
12 to 24 y	64 (12.3)	25 (9.7)	25 (17.0)	14 (12.0)	
25 to 44 y	207 (39.7)	87 (33.9)	65 (44.2)	55 (47.0)	
45 to 64 y	134 (25.7)	66 (25.7)	31 (21.1)	37 (31.6)	
≥65 y	52 (10.0)	38 (14.8)	6 (4.1)	8 (6.8)	
Male	279 (53.6)	136 (52.9)	85 (57.8)	58 (49.6)	0.394
Pain score, among ≥12 y (*N* = 457)^a^	6 (4; 8)	5 (2; 7)	7 (6; 8)	6 (4; 8)	<0.001
Presenting with symptoms	489 (93.9)	228 (88.7)	147 (100)	114 (97.4)	<0.001
Diagnosis					<0.001
Other types of inflammation and pain	192 (36.9)	118 (45.9)	29 (19.7)	45 (38.5)	
Periapical pathologies	117 (22.5)	39 (15.2)	51 (34.7)	27 (23.1)	
Pulp pathologies	117 (22.5)	42 (16.3)	40 (27.2)	35 (29.9)	
Traumas	22 (4.2)	7 (2.7)	14 (9.5)	1 (0.9)	
Other emergencies	73 (14.0)	51 (19.8)	13 (8.8)	9 (7.7)	
Seniority of dentist in charge of teleconsultation					0.008
First-year residents (*n* = 5)	113 (21.7)	53 (20.6)	26 (17.7)	34 (29.1)	
Second- and third-year residents (*n* = 17)	290 (55.7)	153 (59.5)	74 (50.3)	63 (53.8)	
Senior dentists (*n* = 22)	118 (22.6)	51 (19.8)	47 (32.0)	20 (17.1)	
Antibiotics use	57 (10.9)	25 (9.7)	19 (12.9)	13 (11.1)	0.611
Analgesics and NSAID use					<0.001
None	219 (42.0)	141 (54.9)	40 (27.2)	38 (32.5)	
Analgesics alone	189 (36.3)	87 (33.9)	57 (38.8)	45 (38.5)	
NSAID alone	38 (7.3)	11 (4.3)	12 (8.2)	15 (12.8)	
Dual therapy (analgesics and NSAIDs)	75 (14.4)	18 (7.0)	38 (25.9)	19 (16.2)	
COVID-19 at 1-mo follow-up^b^					0.618
No	485 (93.6)	242 (94.2)	136 (93.8)	107 (92.2)	
Suspected	29 (5.6)	13 (5.1)	7 (4.8)	9 (7.8)	
Confirmed	4 (0.8)	2 (0.8)	2 (1.4)	0 (0.0)	

COVID-19, coronavirus disease 2019; NSAID, nonsteroidal anti-inflammatory drug.

a 6 missing values (1.2%).

b 3 missing values (0.6%).

A binary logistic regression was conducted to identify the factors associated with patient admission decision at T0. Admission of patients aged ≥12 y was significantly associated with age, pain score, diagnosis, dentist seniority, and analgesics and NSAID use (*P* < 0.05) (Table 2). The variables that were not significantly associated with the outcome (*P* < 0.05) in the multivariate regression were not included in Table 2. No variable was significant in the multivariate model for patients <12 y (*N* = 64, data not shown).

**Table 2.** Factors Associated in Multivariable Binary Logistic Regression with Admission Decision at T0 (*N* = 457) and with Management Failure Within the Remote Management Group (*N* = 314), among People Aged ≥12 y.

Variable	Factors Associated with Admission Decision at T0		Factors Associated with Management Failure within Remote Management Group	
	OR (95% CI)	*P* Value	OR (95% CI)	*P* Value
Age, per 10 y	0.81 (0.69; 0.94)	0.005		
Male				
Pain score	1.32 (1.18–1.49)	<0.001		
Diagnosis				
Other types of inflammation and pain	0.74 (0.40–1.37)	0.339	0.34 (0.18–0.64)	<0.001
Periapical pathologies	2.01 (1.08–3.80)	0.029	0.51 (0.24–1.07)	0.079
Pulp pathologies	1.00		1.00	
Traumas and other emergencies	5.35 (2.00–15.08)	<0.001	0.23 (0.09–0.59)	0.003
Seniority of dentist in charge of teleconsultation				
First-year residents	1.00		1.00	
Second- and third-year residents	1.40 (0.79–2.55)	0.258	0.62 (0.34–1.11)	0.103
Senior dentists	2.68 (1.38–5.29)	0.004	0.39 (0.17–0.86)	0.022
Antibiotics use				
Analgesics and NSAID use				
None	1.00		1.00	
Analgesics alone	2.03 (1.00–4.31)	0.056	1.02 (0.55–1.91)	0.942
NSAID alone	2.32 (0.88–6.16)	0.088	3.17 (1.20–8.52)	0.020
Dual therapy (analgesics and NSAIDs)	4.64 (2.07–10.82)	<0.001	1.84 (0.76–4.44)	0.173

NSAID, nonsteroidal anti-inflammatory drug; OR, odds ratio.

### Efficacy of On-Site and Remote Management Strategies

The proportion of successful management was 71.8% and 90.2% for remote and on-site groups, respectively.

The factors associated with admission (management failure) within the remote management group in binary logistic regression are presented in Table 2. The model was not applied for children <12 y since very few (3/44, 6.8%) experienced failure. In patients aged ≥12 y, diagnosis was significantly associated with failure with a higher odds ratio (OR) for pulp pathologies compared with the other diagnosis categories. A significant effect of dentist seniority was observed, with a lower OR for senior dentists compared to junior categories. Finally, patient medication with NSAIDs at T0 was also predictive of management failure.

Regarding management failure within the on-site management group, the model was not applied for children <12 y since none of those admitted at T0 required readmission (*n* = 0/20). For patients aged ≥12 y, none of the variables were significantly associated with failure in the multivariate model (*P* > 0.05) (*n* = 16/143, 11.2%). The large majority of management failures in both groups occurred ≤1W (68.8% for on-site and 74.2% for remote management) (Fig. 2b).

### Evolution of Symptomatology and Pain Score

The successful management cases that did not require any additional treatment or advice were assessed from T0 to 1W and from 1W to 1M for the evolution of symptomatology (*N* = 187 for remote management and *N* = 127 for on-site management) and pain score (*N* = 154 for remote management and *N* = 108 for on-site management). The evolution of symptomatology for the majority of those cases was favorable at 1W and 1M (Table 3), with most patients reporting symptoms resolution or improvement. The percentage of status quo or tolerable increase in symptoms was lower in the on-site group than in the remote group (Table 3). Similarly, the reduction of pain scores at 1W and 1M was significant in both groups but even more so in the on-site group (Fig. 2c).

**Table 3.** Evolution of Symptomatology at 1 wk and 1 mo of Successful Management Cases That Did Not Require Any Additional Treatment or Advice (*n* = 187 in the Remote Management Group and *n* = 127 in the On-Site Management Group).

Evolution of Symptoms at 1 mo	Evolution of Symptoms at 1 wk				
	Status Quo with No Symptom, *n* (%)(*n* = 19)	Symptoms Resolution, *n* (%)(*n* = 68)	Improvement of Symptoms, *n* (%)(*n* = 69)	Status Quo, *n* (%)(*n* = 23)	Tolerable Increase in Symptoms, *n* (%)(*n* = 8)
Remote management group (*n* = 187)					
Status quo with no symptom	19 (100.0)				
Symptoms resolution			24 (34.8)	9 (39.1)	2 (25.0)
Improvement of symptoms			25 (36.2)	4 (17.4)	2 (25.0)
Status quo		61 (89.7)	14 (20.3)	9 (39.1)	4 (50.0)
Tolerable increase in symptoms	0 (0.0)	7 (10.3)	6 (8.7)	1 (4.4)	0 (0.0)
On-site management group (*n* = 127)	(*n* = 0)	(*n* = 83)	(*n* = 38)	(*n* = 5)	(*n* = 1)
Status quo with no symptom					
Symptoms resolution			17 (44.7)	1 (20.0)	0 (0.0)
Improvement of symptoms			13 (34.2)	1 (20.0)	1 (100.0)
Status quo		78 (94.0)	3 (7.9)	3 (60.0)	0 (0.0)
Tolerable increase in symptoms		5 (6.0)	5 (13.2)	0 (0.0)	0 (0.0)

### COVID-19 Infection among Patients and Practitioners

The number of patients diagnosed with COVID-19 within the 1 mo following T0 was very low (0.8% of the whole sample) without any significant differences between groups (*P* = 0.618, Table 1).

Among the 44 practitioners, none tested positive for COVID-19 and 7 (15.9%) were qualified as suspected cases over the patient inclusion period or within the month thereafter.

## Discussion

The first major finding was the relative success of our triage-based management strategy, which allowed a high reduction in patient flow since approximately half (49.3%) could be managed remotely without the need for emergency admission within the first month following initial contact. The COVID-19 situation has created unique circumstances in which a dental emergency management strategy involving triage and prospective follow-up was both possible and necessary. Unlike the context of military scenarios or dentistry in remote locations, access to dental care in the current pandemic was possible but restricted as recommended in many countries. Here, the stability of patient symptoms and pain score observed (for 1M) following the emergency management has enabled safe and effective reduction of the demand within emergency clinics.

The second major finding was the high proportion of successful management for both remote (71.8%) and on-site (90.2%) strategies. The high success rate of on-site emergency management could be expected since it was performed in accordance with best-practice recommendations. On the contrary, the relatively high success rate of the new approach of remote management, associated with favorable evolution of symptomatology and pain score, was very encouraging. The stability of patient relief between 1W and 1M, despite the high initial level of pain, illustrates the lasting effect of the treatments provided. This was the case with both on-site and remote strategies, which underlines that most dental emergencies involve the management of acute phases (<1W) by means of advice and/or drug prescription or local treatment. The data also show that definitive treatments (if indicated) can be safely postponed for at least 1 mo.

The results of the multivariate analysis suggest that the success of remote management could be further improved by slightly revising admission guidelines regarding patients using NSAIDs and those with pulp pathologies, given the higher management failure risk in these patients. The admission decision in patients ≥12 y (constituting most of the sample) was mostly motivated by pain score, diagnosis, analgesics and NSAID use, patient age, and dentist seniority. The first 3 variables confirm a good adherence of the practitioners to the suggested and nonbinding guidelines, whereby admissions increased with intense pain, combined use of analgesics and NSAIDs, trauma, and periapical pathologies. The need for minor adjustments in admission decision making may simply rely on the experience level of each practitioner in the group, since more senior personnel tended to admit more and were associated with a lower proportion of management failure among the nonadmitted patients. Therefore, experience proved useful for interpretation of the guidelines. It may have been expected that the protective effect of patient age regarding admission and the higher risk of severe COVID-19 forms among older patients (Davies et al. 2020) would have influenced practitioners’ decisions for admission. However, this was not the case, since age was not associated with management failure risk in the nonadmitted group in the multivariate model. The most likely explanation may have been related to the decreased requirement for emergency dental treatments with older patients, which has also been previously reported (Currie et al. 2017).

An apparent strength of this work was the large sample size (*n* = 521) and the high global follow-up rate (91.4%). Another important aspect was that such a prospective evaluation at similar timepoints of remote and on-site emergency management strategies has, to our knowledge, never been reported before. Nevertheless, there were perhaps limited reasons for conducting such a study prior to the onset of the current global pandemic. This investigation has provided a new perspective on dental emergency management linked to the degree of emergency rather than the severity of the pathology. Contrary to the latter, the former is compatible with telediagnosis, which is mostly based on patient history, with limited or no clinical or imaging elements available.

The number of practitioners involved and the nonbinding characteristics of the guidelines enabled the applicability of the study in a large clinical facility. No calibration was performed regarding admission decision making due to the urgency of the situation and to allow a more clinically representative situation where dentists with different levels of experience maintained a certain freedom of interpretation. This degree of freedom was also consistent with the American Dental Association (2020) recommendations. A limitation of our work was the lack of a comparative condition for the triage strategy. Other triage criteria could be considered in the future (e.g., based on practical aspects such as the aerosol generation required for some treatments). Another possible limitation of the current work was the restriction of medication recommendations to paracetamol in our guidelines, whereas the most efficient medication for acute pain management is a combination of ibuprofen and paracetamol (Moore et al. 2015). However, we refrained from using NSAIDs in our strategy since there were (at the time, April 2020) concerns about their use in COVID-19 patients. Subsequent studies have refuted the avoidance of NSAIDs of COVID-19–positive patients (de Girolamo et al. 2020; FitzGerald 2020; Little 2020). Regardless, the association of NSAID use with failure among nonadmitted patients at T0 (Table 2) underlines either an insufficient response of certain pathologies to this medication or their use in inflammatory conditions that are already too advanced and therefore requiring local treatment.

Comparison of the data presented in this study with other previous research is impossible due to the novelty of the disease and the absence of similar works. Unlike the current study, the recommendations for dental emergency management available to date have not included any clinical data regarding patient outcomes (Ather et al. 2020; Izzetti et al. 2020). Furthermore, our work highlighted the usefulness of teledentistry in the context of dental emergency management, beyond the routine consultations considered so far (Alabdullah and Daniel 2018; Estai et al. 2018). In such case, and as reported in a recent pilot study (Giudice et al. 2020), clinical pictures taken by patients and shared with their practitioner may allow a more objective evaluation, notably for the presence of swelling and suspicion of abscesses, and a more reliable follow-up of the pathology over time. Similarly, the widely used and easily understood numerical rating scale for pain assessment (Sharav et al. 1984; Seymour et al. 1985) was a useful tool for both admission decision making and follow-up. Finally, high expectation of pain associated with dental pathologies was shown to generate anxiety, which may have affected the patients’ ability to process clinical information (Eli et al. 2008).

Since the infectious COVID-19 status of patients was not tested at T0, it is not possible to formally conclude the safety of the present strategy with regards to the risk of cross-contamination. However, there is a likelihood that a proportion of treated patients was positive and asymptomatic (Bohmer et al. 2020; Cheng et al. 2020; Ganyani et al. 2020; Gudbjartsson et al. 2020). In this context, it was interesting to observe that the proportion of positive COVID-19 cases 1M after initial contact was very low, both in the remote and on-site management groups, as well as among the practitioners. Hence, this is in line with the available evidence showing that the use of appropriate personal protection equipment and hygiene measures when performing dental treatments, including those generating aerosols, can prevent cross-infection (Meng, Ma, et al. 2020).

The proposed triage-based emergency management strategy investigated here has provided an effective compromise between limiting patient admission and ensuring their pain control and symptom relief. The information and advice provided remotely need to be combined with a systematic follow-up, which proved significantly beneficial not only for verification of the treatment efficacy but also to reassure the patient. The strategy responded to several points made by a recent World Health Organization (2020) report and can be useful in future situations where regulation of the emergency patient flow is required.

## Author Contributions

J. Beauquis, J.G. Leprince, contributed to conception, design, data acquisition, analysis, and interpretation, drafted and critically revised the manuscript; A.E. Petit, contributed to design, data analysis, and interpretation, critically revised the manuscript; V. Michaux, V. Sagué, contributed to data acquisition and interpretation, drafted the manuscript; S. Henrard, contributed to data analysis and interpretation, drafted and critically revised the manuscript. All authors gave final approval and agree to be accountable for all aspects of the work.

## Supplemental Material

sj-pdf-1-jdr-10.1177_0022034521990314 – Supplemental material for Dental Emergencies Management in COVID-19 Pandemic Peak: A Cohort Study Click here for additional data file. Supplemental material, sj-pdf-1-jdr-10.1177_0022034521990314 for Dental Emergencies Management in COVID-19 Pandemic Peak: A Cohort Study by J. Beauquis, A.E. Petit, V. Michaux, V. Sagué, S. Henrard and J.G. Leprince in Journal of Dental Research
