# Supplementary material for: Dental Emergencies Management in COVID-19 Pandemic Peak: A Cohort Study
Source: J Dent Res. 2021 Feb 4;100(4):352–60. doi: 10.1177/0022034521990314 (PMC7868351; doi:10.1177/0022034521990314)
Supplement: sj-pdf-1-jdr-10.1177_0022034521990314 – Supplemental material for Dental Emergencies Management in COVID-19 Pandemic Peak: A Cohort Study [file sj-pdf-1-jdr-10.1177_0022034521990314.pdf]

Appendix

Title : Dental emergencies management in COVID-19 pandemic peak: a cohort study

Authors names: Julien Beauquis [1,2*], Anne-Elisabeth Petit [3,4], Valentin Michaux[1,2], Vincent Sagué[2,5], Séverine Henrard[4,6], Julian G Leprince[1,2]

COVID-19 in the present cohort

We decided that confirmed COVID-19 cases (RT- PCR, serology or chest CT-scan) or suspected cases (fever > 37.5°C, respiratory symptoms compatible with COVID-19 such as dry cough or dyspnea) should be managed in accordance with the established guidelines, hence remotely if possible; if local treatment was required, it should be performed in the COVID-19 area of the main hospital (n=0).

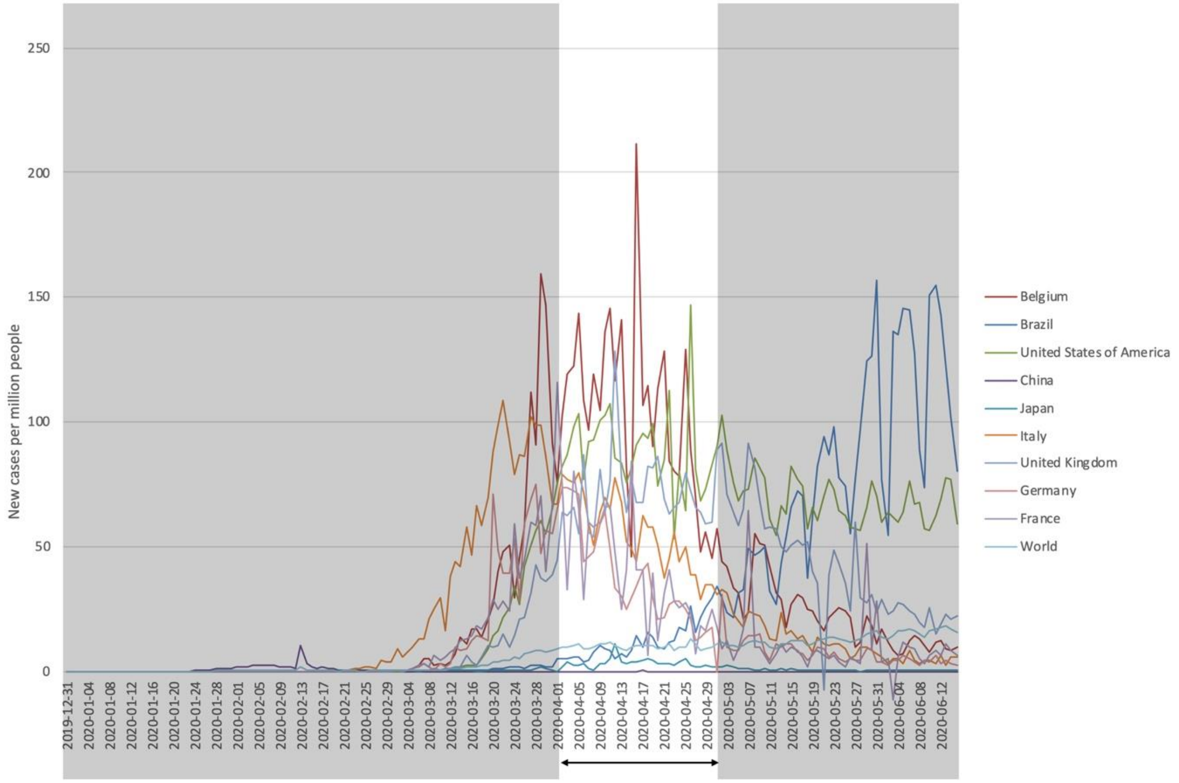

Appendix Figure 1 – (a) Evolution of the number of daily new COVID-19 cases during the period of investigation in Belgium, compared to other key countries (https://ourworldindata.org/coronavirus-data; [last accessed august 2020]))

Collected variables and grouping for categorial variables

Appendix Table 1 – Types of individual diagnoses and their categories

**Pulp pathologies**
Tooth hypersensitivity<br>Reversible pulpitis<br>Irreversible pulpitis<br>Root pulpitis<br>Unclear pulp pain
**Periapical pathologies**
Acute apical periodontitis<br>Acute apical periodontitis with abscess<br>Chronic apical periodontitis<br>Chronic apical periodontitis with acute exacerbation<br>Chronic apical periodontitis with acute exacerbation and abscess<br>Undefined periapical pathology without abscess<br>Undefined periapical pathology with abscess<br>Endodontic periodontal lesion
**Other types of inflammation and pain**
Abscess without clear origin<br>Gingivitis<br>Chronic periodontitis<br>Chronic aggressive periodontitis<br>Periodontal abscess<br>Pain without clear origin<br>Occlusal overload (periodontal ligament inflammation)<br>Mucosal ulceration<br>Pericoronitis<br>Unidentified mucosal pathology<br>Salivary lithiasis<br>White mucosal lesion

Alveolitis<br><br>Temporomandibular disorder<br><br>3
**Traumas**
Trauma without pulp symptoms<br><br>Trauma with pulp symptoms<br><br>Avulsion of deciduous tooth<br><br>Avulsion of permanent tooth<br><br>Luxation of deciduous tooth<br><br>Trauma with pulp exposure<br><br>Mucosal lesion<br><br>Multiple lesions
**Other emergencies**
Tooth fracture requiring removal of the fragment without pulp symptoms<br><br>Loss of one or more portions of the tooth without pulp symptoms<br><br>Loss of filling (without pulp symptoms)<br><br>Implant loss<br><br>Halitosis<br><br>Error of admission<br><br>Loss of orthodontic braces<br><br>Patient follow-up<br><br>Crown/Bridge loss<br><br>Crown on loose implant<br><br>Prosthesis fracture<br><br>Poorly fitted prosthesis<br><br>Fracture of a fixed element (crown or bridge)<br><br>Tooth eruption<br><br>Dental advice

Statistical analysis

Continuous variables are presented as medians [P_{25}; P_{75}] and were compared between groups using a Kruskal Wallis test. Categorical variables are presented as number of proportions and are compared between groups using Pearson's Chi² test, Pearson's Chi² test with Yates continuity correction, or Fisher-Freeman-Halton test depending on the condition of validity of each test. The Pearson's chi-squared test was performed when the minimal expected value was ≥5 in the crosstable, the Pearson's chi-squared test with Yates continuity correction was used when the minimal expected value was between 3 and 5, and the Fisher-Freeman-Halton test was used when the minimal expected value was <3.

Wilcoxon signed-rank test was used to compare pain score evolution between 1W and T0, and between 1M and 1W in paired patients.

Factors associated with admission at T0 among all patients, with failure of the management among the two groups were assessed using a binary logistic regression. All variables with a p-value < 0.15 in univariate analysis (see additional tables) were candidate for the multivariate model. In the multivariate model, a stepwise selection using Akaike Information Criterion was applied to select the final multivariate model. Finally, a goodness-of-fit of the model was assessed using the Hosmer and Lemeshow test and multicollinearity was assessed using the variance inflation factor.

All analyses were performed using R software version 3.3.1. a p-value < 0.05 was considered statistically significant.

Additional tables: results of the univariate models

Appendix Table 2. Factors associated with admission decision at T0 among people aged ≥12 years (N=457) in the univariate model

	Univariate model	
**Variables**	**OR (95%CI)**	**p-value**
**Among people aged ≥ 12 years (N=457)**		
Age, per 10 years	0.76 (0.66; 0.86)	<0.001
Male	1.18 (0.79; 1.75)	0.423
Pain score	1.33 (1.22; 1.46)	<0.001
Diagnosis		
Other types of inflammation and pain	0.45 (0.26; 0.78)	0.004
Periapical pathologies	1.66 (0.96; 2.89)	0.071
Pulp pathologies	1.00	
Traumas and other emergencies	0.62 (0.31; 1.20)	0.159
Seniority of dentist in charge of teleconsultation		
First year residents	1.00	
2nd and 3rd year residents	1.21 (0.72; 2.06)	0.474
Senior dentists	2.58 (1.44; 4.70)	0.002
Antibiotics use	1.72 (0.97; 3.02)	0.062
Analgesics and NSAID use		
None	1.00	
Analgesics alone	2.93 (1.74; 5.10)	<0.001
NSAID alone	3.83 (1.73; 8.42)	<0.001
Dual therapy (analgesics and NSAID)	7.89 (4.23; 15.14)	<0.001

NSAID: Nonsteroidal anti-inflammatory drug

Appendix Table 3. Factors associated with *remote* management failure among people aged ≥12 years (N=314) in the univariate model

	Univariate model	
**Variables**	**OR (95%CI)**	**p-value**
Age, per 10 years	0.87 (0.75; 1.01)	0.071
Male	1.01 (0.63; 1.64)	0.958
Pain score	1.10 (1.01; 1.20)	0.041
Diagnosis		
Other types of inflammation and pain	0.39 (0.21; 0.71)	0.002
Periapical pathologies	0.57 (0.27; 1.18)	0.135
Pulp pathologies	1.00	
Traumas and other emergencies	0.22 (0.09; 0.51)	<0.001
Seniority of dentist in charge of teleconsultation		
First year residents	1.00	
2nd and 3rd year residents	0.67 (0.39; 1.17)	0.156
Senior dentists	0.53 (0.24; 1.10)	0.096
Antibiotics use	0.68 (0.28; 1.50)	0.364
Analgesics and NSAID use		
None	1.00	
Analgesics alone	1.42 (0.82; 2.46)	0.210
NSAID alone	3.37 (1.36; 8.48)	0.009
Dual therapy (analgesics and NSAID)	2.40 (1.07; 5.36)	0.032
Medication advice given	1.07 (0.65; 1.80)	0.780
Non-medication advice given	0.70 (0.40; 1.21)	0.191

NSAID: Nonsteroidal anti-inflammatory drug

Appendix Table 4. Factors associated with *on-site* management failure among people aged ≥12 years (N=143) in the univariate model

Variables	Univariate model OR (95%CI)	p-value
Age, per 10 years	1.04 (0.72; 1.46)	0.830
Male	0.76 (0.27; 2.20)	0.612
Pain score	1.11 (0.89; 1.44)	0.401
Diagnosis		
Other types of Inflammation and pain	4.37 (0.97; 30.95)	0.079
Periapical pathologies	2.23 (0.48; 15.88)	0.342
Pulp pathologies	1.00	
Traumas and other emergencies	1.03 (0.05; 11.49)	0.982
Antibiotics use	2.58 (0.75; 8.01)	0.110
Analgesics and NSAID use		
None	1.00	
Analgesics alone	1.12 (0.24; 8.09)	0.891
NSAID alone	2.62 (0.38; 22.15)	0.326
Dual therapy (analgesics and NSAID)	1.38 (0.27; 10.23)	0.713
Medication advice given	1.12 (0.39; 3.21)	0.835
Non medication advice given	1.01 (0.36; 3.00)	0.979

NSAID: Nonsteroidal anti-inflammatory drug
